# Implementing a community vector collection strategy using xenomonitoring for the endgame of lymphatic filariasis elimination

**DOI:** 10.1186/s13071-018-3260-3

**Published:** 2018-12-27

**Authors:** Sellase Pi-Bansa, Joseph Harold Nyarko Osei, Joannitta Joannides, Maame Esi Woode, David Agyemang, Elizabeth Elhassan, Samuel Kweku Dadzie, Maxwell Alexander Appawu, Michael David Wilson, Benjamin Guibehi Koudou, Dziedzom Komi de Souza, Jürg Utzinger, Daniel Adjei Boakye

**Affiliations:** 1 Swiss Tropical and Public Health Institute, Basel, Switzerland; 2 University of Basel, Basel, Switzerland; 3 Noguchi Memorial Institute for Medical Research, College of Health Sciences, University of Ghana, Legon, Ghana; 4 Department of Animal Biology and Conservation Science, University of Ghana, Legon, Ghana; 5 Liverpool School of Tropical Medicine, Liverpool, UK; 6 SightSavers International, Ghana Office, Accra, Ghana; 7 Centre Suisse de Recherches Scientifiques en Côte d’Ivoire, Abidjan, Côte d’Ivoire

**Keywords:** Xenomonitoring, Validation, Lymphatic filariasis, *Wuchereria bancrofti*, Community vector collectors

## Abstract

**Background:**

The global strategy for elimination of lymphatic filariasis is by annual mass drug administration (MDA). Effective implementation of this strategy in endemic areas reduces *Wuchereria bancrofti* in the blood of infected individuals to very low levels. This minimises the rate at which vectors successfully pick microfilariae from infected blood, hence requiring large mosquito numbers to detect infections. The aim of this study was to assess the feasibility of using trained community vector collectors (CVCs) to sample large mosquito numbers with minimal supervision at low cost for potential scale-up of this strategy.

**Methods:**

CVCs and supervisors were trained in mosquito sampling methods, i.e. human landing collections, pyrethrum spray collections and window exit traps. Mosquito sampling was done over a 13-month period. Validation was conducted by a research team as quality control for mosquitoes sampled by CVCs. Data were analyzed for number of mosquitoes collected and cost incurred by the research team and CVCs during the validation phase of the study.

**Results:**

A total of 31,064 and 8720 mosquitoes were sampled by CVCs and the research team, respectively. We found a significant difference (*F*_(1,13)_ = 27.1606, *P* = 0.0001) in the total number of mosquitoes collected from southern and northern communities. Validation revealed similar numbers of mosquitoes sampled by CVCs and the research team, both in the wet (*F*_(1,4)_ = 1.875, *P* = 0.309) and dry (*F*_(1,4)_ = 2.276, *P* = 0.258) seasons in the southern communities, but was significantly different for both wet (*F*_(1,4)_ = 0.022, *P* = 0.005) and dry (*F*_(1,4 )_ = 0.079, *P* = 0.033) seasons in the north. The cost of sampling mosquitoes per season was considerably lower by CVCs compared to the research team (15.170 *vs* 53.739 USD).

**Conclusions:**

This study revealed the feasibility of using CVCs to sample large numbers of mosquitoes with minimal supervision from a research team at considerably lower cost than a research team for lymphatic filariasis xenomonitoring. However, evaluation of the selection and motivation of CVCs, acceptability of CVCs strategy and its epidemiological relevance for lymphatic filariasis xenomonitoring programmes need to be assessed in greater detail.

## Background

Lymphatic filariasis is a neglected tropical disease caused by infection with the parasitic worms *Wuchereria bancrofti*, *Brugia malayi* and *B. timori*, all of which are transmitted by mosquitoes [1]. There are various species of mosquitoes implicated in the life-cycle of the parasites, mainly of the genera *Aedes*, *Anopheles*, *Coquillettidia*, *Culex* and *Mansonia* [2]. These species differ in their biology, distribution, ecology and transmission potential. The Global Programme to Eliminate Lymphatic Filariasis (GPELF) was launched in 2000 with the goal to eliminate lymphatic filariasis by interrupting transmission through MDA and reducing morbidity and disability [3]. The adopted MDA strategy is annual treatment with a single dose of albendazole in combination with either ivermectin or diethylcarbamazine (DEC) for 4–6 years [4]. However, a combination of these three drugs (IDA) was approved in 2017 by the World Health Organization (WHO) to be used only in regions non-endemic for onchocerciasis and loiasis [5, 6]. The GPELF has achieved great success since its inception by preparing guidelines in all endemic regions and facilitating the implementation and scaling up of lymphatic filariasis MDA in endemic countries. Indeed, by the end of 2015 over 6.2 billion cumulative treatments were distributed [7], resulting in strong declines of microfilaraemia (36.45 million), hydrocele (19.43 million) and lymphedema (16.68 million) in 2013 [8]. Of the 73 endemic countries, 18 countries moved into post-transmission surveillance, following successful transmission assessment surveys (TAS) [7]. Despite this progress, it will be difficult for most of endemic countries to become verified as free of transmission or having entered the post-intervention surveillance phase by 2020 [1], as recognised recently at the Expanded Special Project for Elimination of Neglected Tropical Diseases (ESPEN) in Kigali.

Following successful MDA implementation, the prevalence of infection falls below or equals the critical cut-off threshold for interrupting transmission by various vectors. For *Anopheles* and *Culex*, the threshold is < 2% antigenaemia prevalence. For *Aedes*, the threshold is < 1% antigenaemia prevalence [9]. This poses significant challenges to xenomonitoring because at such low levels of infection, large numbers of mosquitoes must be analysed in order to assess whether transmission of the disease in the vectors has indeed been halted, which is costly [10, 11]. Additionally, longitudinal entomological monitoring strategies rely on trained specialist technical staff who are usually limited in both their geographical scope and the frequency of sampling at any survey location [12]. To that end, there is a need to employ new strategies that can effectively allow the collection of large numbers of mosquitoes, at greatly reduced cost, while exploring the temporal and spatial patterns of lymphatic filariasis vector transmission indices.

The present study was undertaken to address the need for sampling large numbers of mosquitoes for xenomonitoring purposes, at low costs [1]. Hence, we determined the ability of community collectors to successfully collect mosquitoes with minimal supervision from a research team, including costs in order to assess the feasibility of implementing this approach on a large scale. To this end, we determined a concept of using trained community vector collectors (CVCs) for the collection of mosquitoes, similar to community drug distributors (CDDs) implementing MDA.

## Methods

### Study sites

**Fig. 1:**
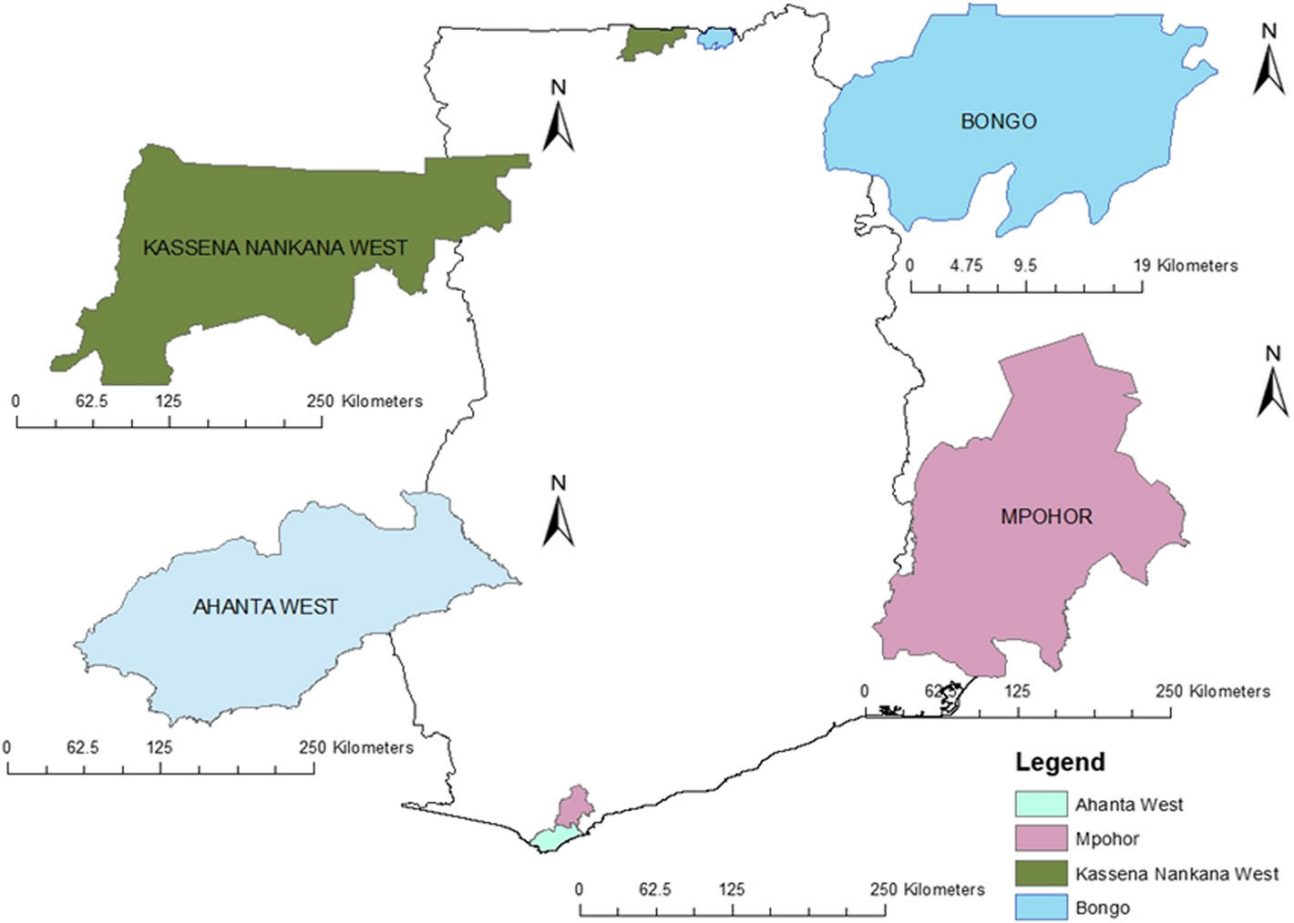
Map showing lymphatic filariasis study areas from northern and southern districts, Ghana

Four districts were selected in lymphatic filariasis-endemic areas of Ghana. Two districts from the north, namely Kassena Nankana West (0°10'N, 10°50'W) and Bongo (0°45'N, 10°50'W) were identified as study sites (Fig. 1). The reported population sizes for the Bongo and Kassena Nankana West districts by the Ghana Statistical Service for the year 2010 were 84,545 [13] and 70,667 [14], respectively. Inhabitants located in these two districts are mostly farmers involved in growing crops, rearing livestock and fish farming [15]. Climate in the north is characterised by wet and dry seasons, with average rainfall ranging between 645 and 1250 mm [15]. The average temperature and relative humidity are 15–45°C and 30–80%, respectively [15]. Additionally, two districts from the south, namely Ahanta West (4°84'N, 2°02'W) and Mpohor (4°05'N, 1°54'W) were selected. In the year 2010, the population sizes recorded for Ahanta West and Mpohor districts were 106,215 and 42,923, respectively [16, 17]. Indigenes in both districts are mostly fishermen/fishmongers and farmers [15]. Ahanta West and Mpohor districts lie within the high rainfall zone in Ghana, with average rainfall of 1600 mm per year [15]. The average temperature and humidity in the south are 20–34 °C and 75–80%, respectively [15]. The southern districts are characterised by rainforests, mangrove zones and high precipitation [18]. The northern districts fall within the arid Sudan savannah zone [19]. Data from the 2016 annual report of the Ghana Health Service (GHS) indicate malaria to be endemic in all study districts [20]. However, lymphatic filariasis is endemic in all districts except Mpohor [20].

### Community engagement and training of vector collectors

Community engagement was undertaken to inform the district health administration, community chiefs/elders and community members. Following the community engagement, the community elders were invited to identify individuals who will serve as vector collectors. The elders were asked to identify 9 volunteers, either male or female, 18 years-old and above, with formal or informal education in the community. However, the selection criterion for the supervisor was to identify an individual who had at least completed junior high school. Furthermore, no experience of prior mosquito collection was required to be selected as a CVC. The selected community volunteers and supervisors were trained in specific mosquito collection procedures. These included pyrethrum spray collection, window exit traps and human landing collections [1]. The use of the three methods was to maximise the number of mosquitoes collected for xenomonitoring purposes. The supervisors were also trained on the best ways to package, store and ship collected mosquitoes. Mosquitoes sampled using human landing collections were knocked down in their holding cups using cotton wool soaked with chloroform. The knocked down mosquitoes were transferred into a Petri dish and, using a pair of forceps; a maximum of 10 mosquitoes were transferred into labelled Eppendorf tubes. A Pasteur pipette was used to aliquot 200 μl RNA*later* (Life Technologies, Carlsbad, CA, USA) and dispensed into the various Eppendorf tubes containing mosquitoes. The tubes were covered, sealed with strips of parafilm and held in labelled holding racks. Mosquitoes sampled using pyrethrum spray catches and window exit traps were stored in labelled Eppendorf tubes which had their covers pierced. The tubes were then kept in labelled ziplock bags containing silica gel [21].

### Collection of mosquitoes

Following training, collectors were provided with the necessary consumables and supplies to carry out monthly collections. Mosquito collections were done over a period of 13 months from the beginning of July 2015 to the end of July 2016. Collections were done twice each month. For convenience, the CVCs were at liberty to select days appropriate for all of them in the first and second half of the month. Eight community volunteers per district were involved in the collection, with a total of 16 person-days of collection in a month. A supervisor was also identified to ensure that the collections were according to protocol undertaken and serve as the link between the researchers and the vector collectors. The days of collection were left at the discretion of the collectors. In the evening of the sampling night, four window exit traps were fixed in different sections of the communities. Human landing collection was undertaken by two teams of four collectors each [22]. The teams were constituted in order to have two indoor and two outdoor human landing collections, in different sections of the community. Human landing collections were carried out from 21:00 to 05:00 h. Pyrethrum spray collections were done by the same teams in the morning. Up to ten rooms were sampled by all volunteers in the community, on each collection day, using pyrethrum spray collections from 06:00 to 09:00 h. The collected mosquitoes were stored and sent to the researchers by public transport. Every three months the researchers visited the communities to replenish the supplies (i.e. insecticide, tubes, cotton wool, silica gel and RNA*later*) needed for the collection and storage. Outside these periods, payments to the vector collectors were done through bank or mobile money transfers.

### Validation of mosquito sampling survey

A quality control (validation) was implemented for human landing collections and pyrethrum spray collections that are collector and technique-dependent. Validation was also done for window exit traps. This was done on two occasions, in the rainy and dry seasons. Briefly, the research team from Noguchi Memorial Institute for Medical Research made two unannounced visits (one visit per season) to the study communities. In order to validate mosquito sampling done by the CVCs, the Noguchi Memorial Institute for Medical Research team collected mosquitoes from the same households as community vector collectors. The mosquitoes collected were compared with the regular sampling done by the CVCs within the same month. Mosquito collection by the research team was done in the third week of April and July 2016. Two households were selected for mosquito collection using human landing catches and window exit traps each catch night. In the morning, ten households were selected for mosquito collection using the pyrethrum spray method. The time for sampling mosquitoes by the research team using the various sampling techniques was the same as that of the CVCs.

### Analysis of cost data

This work is part of a larger study so only costs explicitly related to the mosquito collection were considered. These costs therefore exclude any costs related to the parasitological analysis of the mosquitoes collected. Costs were split into recurrent and capital costs. Recurrent costs were those that were incurred frequently and include personnel allowances, supplies, transportation, communication, fuel, etc. Capital costs were those investments made in fixed assets, which are used over a longer period and include cost of vehicles, machinery and equipment. Capital costs were annualised. All costs were converted into US Dollars (USD) using the average exchange rate prevailing on the markets during the study period.

### Statistical analysis

Data on costs incurred from the study were entered and analysed using Microsoft Excel 2013. We checked for significant differences of the total number of mosquitoes collected by CVCs from the northern and southern part of Ghana, and between CVCs and the Noguchi Memorial Institute for Medical Research team during validation using *F*-test. *P-*values ≤ 0.05 were considered statistically significant.

## Results

### Mosquito collection

**Fig. 2:**
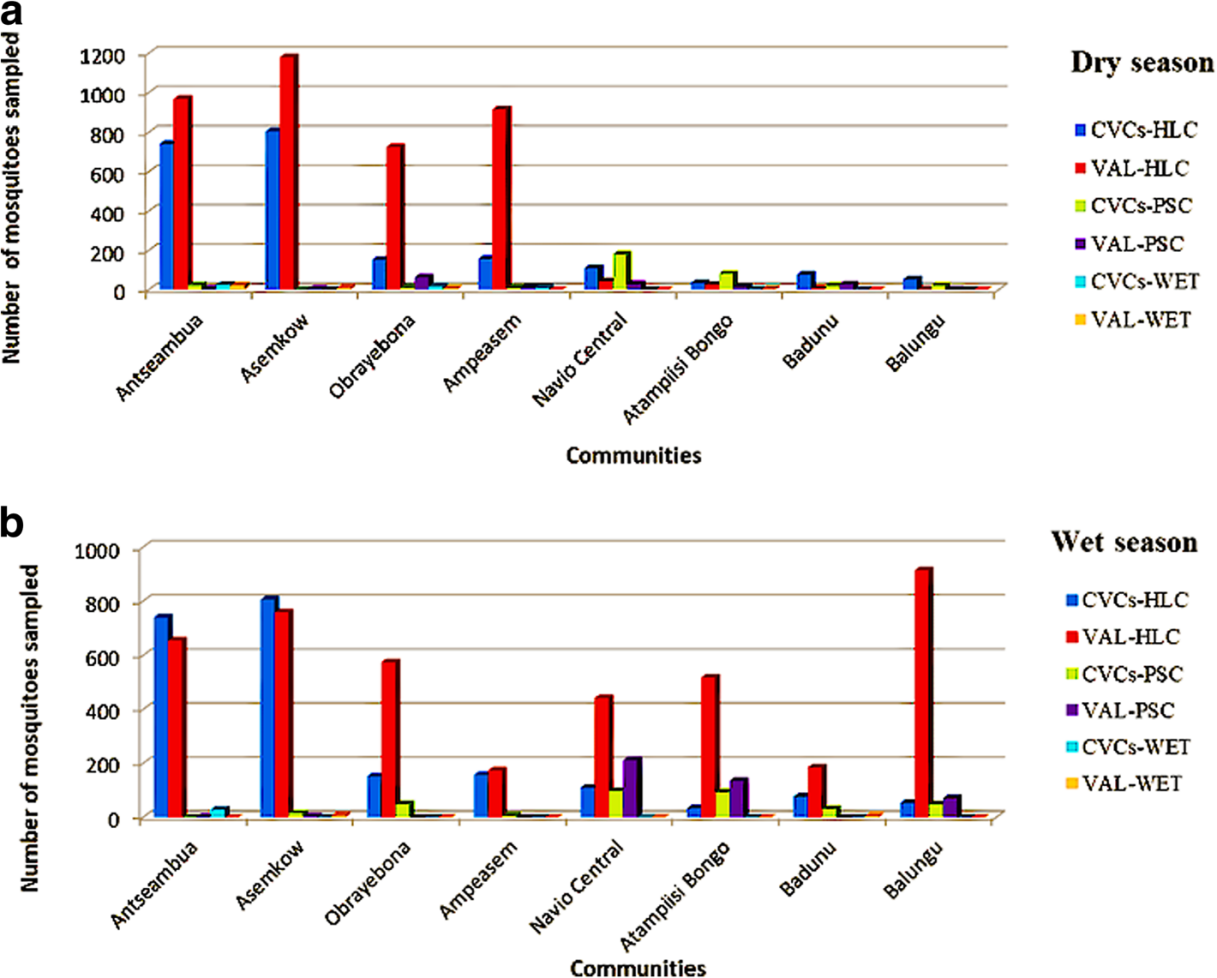
Validation of mosquitoes sampled by CVCs and the research team in the northern and southern communities, Ghana. **a** Validation of mosquitoes sampled by CVCs and the research team in the dry season. **b** Validation of mosquitoes sampled by CVCs and the research team in the rainy season. *Abbreviations*: *VAL* validation, *HLC* human landing collections, *PSC* pyrethrum spray collections, *WET* window exit trap

Over the 13-month study period, a total of 31,064 and 8720 mosquitoes were sampled by CVCs and the Noguchi Memorial Institute for Medical Research team, respectively. Table 1 shows the result of the number of mosquitoes collected by CVCs and the research team during the validation period in the dry and rainy seasons using the three sampling techniques. Mosquito collections were done twice for each month during validation. Human landing collections provided the highest number of mosquitoes caught for xenomonitoring. Higher numbers of mosquitoes were collected by the research team compared to CVCs in the months when both constituencies collected mosquitoes (Fig. 2a, b). However, there was no significant difference in the number of mosquitoes sampled by research team compared to the CVCs for both the rainy (*F*_(1,4)_ = 1.875, *P* = 0.309) and dry (*F*_(1,4)_ = 2.276, *P* = 0.258) seasons in the southern communities. The opposite was observed for the northern communities, where the total number of mosquitoes sampled by the CVCs compared with the research team was significantly different for both the rainy (*F*_(1,4)_ = 0.022, *P* = 0.005) and dry (*F*_(1,4)_ = 0.079, *P* = 0.033) seasons. In the south, human landing collections gave the highest number of mosquitoes in all the communities, whiles pyrethrum spray collections provided a higher number of mosquitoes for communities in the north (Fig. 2a, b). Mosquitoes collected from each of the study sites by the CVCs during the study period are shown in Table 2. Results from Table 2 indicate that the total number of mosquitoes collected by the CVCs was significantly different between the southern coastal communities compared to the northern arid zones (*F*_(1,13)_ *=* 27.1606, *P* < 0.0001).

**Table 1:** Mosquito collection for validation by CVCs and research team in the northern and southern communities, Ghana

Personnel	Dry/Rainy season	North/South	Sampling type (HLC/PSC/WET)	*An. gambiae*	*Culex* spp.	*Ma. uniformis*	*Ma. africana*	*Aedes* spp.	*An. pharoensis*	*An. coustani*	Total
Research team	Dry	South	HLC	3561	198	0	25	0	0	3	3787
			PSC	82	3	0	0	0	0	0	85
			WET	34	1	0	0	0	0	0	35
	Dry	North	HLC	30	42	0	0	0	2	0	74
			PSC	48	21	0	0	0	0	5	74
			WET	1	2	0	0	0	0	0	3
CVCs	Dry	South	HLC	1906	302	31	0	0	0	1	2240
			PSC	38	0	0	0	0	4	0	42
			WET	46	0	0	0	0	8	0	54
	Dry	North	HLC	33	12	0	0	0	0	1	46
			PSC	236	68	0	0	0	0	0	304
			WET	2	0	0	0	0	0	0	2
Research team	Rainy	South	HLC	1984	11	3	166	0	0	0	2164
			PSC	5	1	0	1	0	0	0	7
			WET	7	0	0	0	0	0	0	7
	Rainy	North	HLC	962	1075	2	0	10	7	3	2059
			PSC	376	42	0	0	0	1	1	420
			WET	1	1	0	0	0	0	3	5
CVCs	Rainy	South	HLC	1757	20	4	75	1	0	0	1857
			PSC	64	2	0	5	0	0	0	71
			WET	24	1	0	4	0	0	0	29
	Rainy	North	HLC	123	140	0	1	8	8	2	282
			PSC	186	86	0	0	0	0	0	272
			WET	0	0	0	0	0	0	0	0

*Abbreviations*: *HLC* human landing collections, *PSC* pyrethrum spray collections, *WET* window exit trap, *CVCs* community vector collectors

*Note*: The data represent the number of mosquitoes collected only during the validation period for comparison between the research team and CVCs

**Table 2:** Mosquitoes species collected from the northern and southern communities in Ghana by the CVCs

North/South	District	Community	Mosquito species							
			*An. gambiae*	*Culex* spp.	*Ma. uniformis*	*Ma. africana*	*Aedes* spp.	*An. pharoensis*	*An. coustani*	Total (%)
South	Ahanta West	Asemkow	13,540	69	19	18	2	36	0	13,684 (44.05)
		Antseambua	5340	1152	755	1642	7	0	4	8900 (28.65)
	Mpohor	Ampeasem	2247	5	6	5	4	9	0	2276 (7.33)
		Obrayebona	2356	76	55	14	3	1	3	2508 (8.07)
North	Kassena Nankana West	Navio Central	751	680	6	3	12	2	6	1460 (4.70)
		Badunu	488	199	3	0	32	2	7	731 (2.35)
	Bongo	Atampiisi Bongo	542	200	2	1	23	4	1	773 (2.49)
		Balungu Nabiisi	284	426	1	1	19	0	1	732 (2.36)
Total			25,548	2807	847	1684	102	54	22	31,064 (100)

### Cost estimates

Table 3 shows the result of the breakdown of the total costs incurred by both the research team and CVCs for training and mosquito sampling. The personnel costs include allowances paid to each category of personnel. The personnel costs incurred for the two days of sampling in a month by an individual in the research team and a CVC was 53.73 and 15.17 USD, respectively. Due to financial limitations, the research team from Noguchi Memorial Institute for Medical Research used four instead of eight collectors for sampling during validation. The amount incurred for the two sampling nights in a community by the four research team members, compared to the eight CVCs was 214.92 and 121.36 USD, respectively. The cost estimates for this study are presented in Table 4. The recurrent transportation costs include the cost of fuel, maintenance and repairs undertaken in the field as well as road tolls. The supplies include the pyrethrum insecticide, desiccants and other items that were required for the collection of mosquitoes. Other costs include the cost of communication between the research team and the CVCs, the cost of sending consumables to communities and samples from the communities to the research team using public transport and finally, money transfers. With the exception of when the research team was undertaking a field visit to the communities, the allowances of the CVCs were sent *via* bank or mobile money transfers.

**Table 3:** Training and validation cost for CVCs and research team in the northern and southern communities, Ghana

Activity	Cost of sampling for 2 days in a month			
			Cost (GH¢)	Cost (USD)
Training	Personnel costs	Cost for CVCs	60.00	15.17
		Cost for supervisors	70.00	17.69
		Cost for research team	212.50	53.73
		Cost for driver (research team)	170.00	42.98
	Transportation	Cost for fuel	2713.00	685.96
		Cost for car maintenance	1485.00	375.47
		Cost for road tolls	59.00	14.91
		Cost for motorbike fuel (north)	12.50	3.16
		Cost for motorbike fuel (south)	–	–
Validation (Dry season)	Transportation	Cost for fuel	1733.00	438.17
		Cost for car maintenance	689.75	174.39
		Cost for road tolls	30.50	7.71
		Cost for motorbike fuel (north)	12.50	3.16
		Cost for motor bike fuel (south)	–	–
Validation (Wet season)	Transportation	Cost for fuel	1733.00	438.17
		Cost for car maintenance	689.75	174.39
		Cost for road tolls	30.50	7.71
		Cost for motorbike fuel (north)	12.50	3.16
		Cost for motorbike fuel (south)	–	–

*Note*: Personnel cost is cost per individual per month (2 sampling days), whilst transportation cost is the cost per month for sampling mosquitoes in all study communities during training and validation for wet and dry seasons

**Table 4:** Cost estimates for mosquito sampling process

Cost of xenomonitoring	GH¢	USD
Recurrent costs	105,892.20	26,773.78
Personnel costs	84,520.00	21,370.04
Vector collectors	46,080.00	11,650.87
Supervisors	14,640.00	3701.58
Entomologist	23,800.00	6017.59
Materials and supplies	9968.70	2520.49
Media and IEC operating costs	1510.50	381.91
Transportation operating costs	6299.00	1592.64
Maintenance	2864.50	724.26
Other recurrent costs	729.50	184.45
Capital costs	12,929.40	3269.07
Transport costs	11,470.26	2900.14
Equipment	1459.14	368.93
Total annual cost	118,821.60	30,042.85

**Fig. 3:**
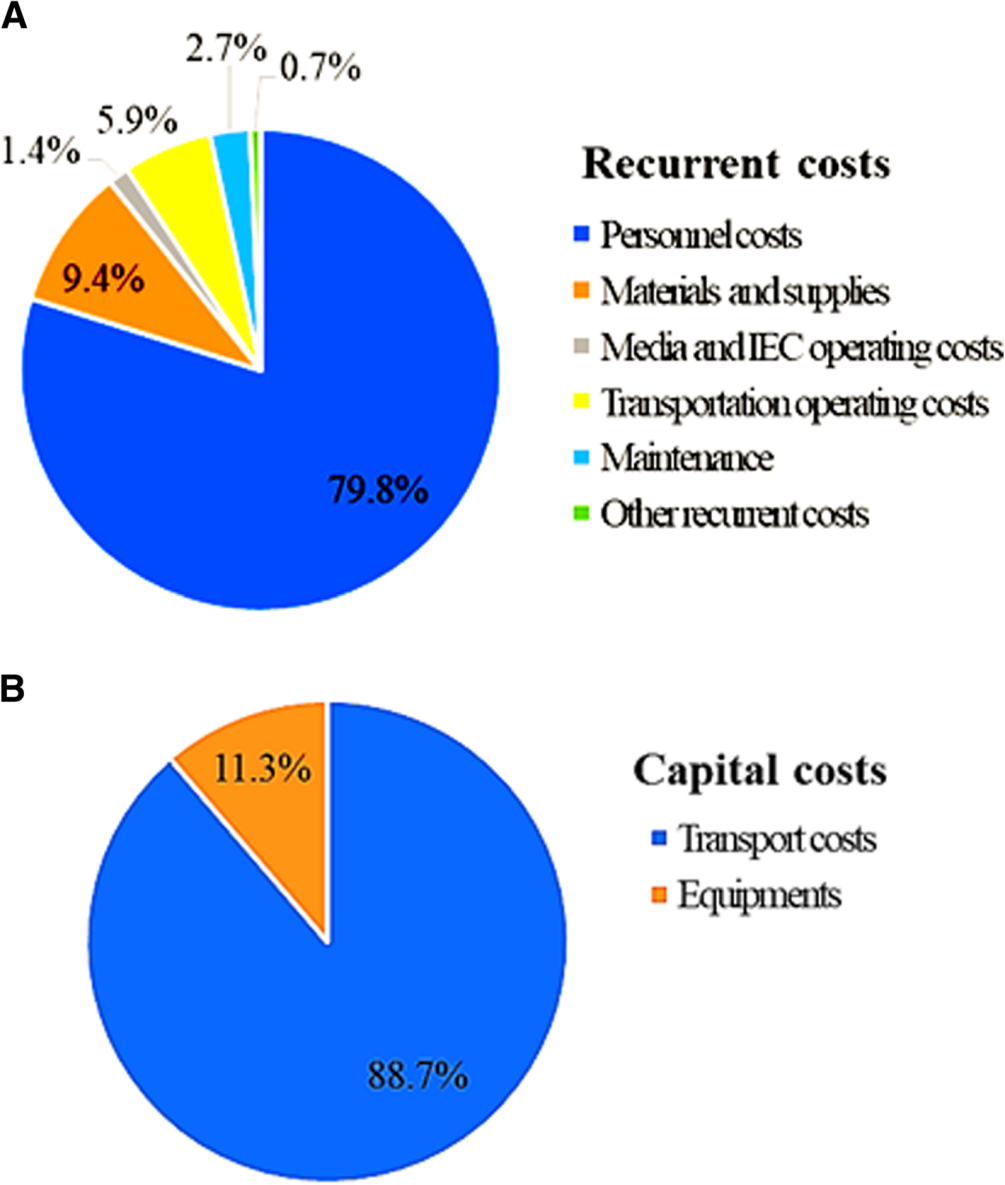
Cost distribution based on type of cost for studies in northern and southern communities, Ghana. **a** The recurrent costs for studies in the northern and southern communities, Ghana. **b** The capital costs for studies in the northern and southern communities, Ghana. *Abbreviation*: IEC, information, education and communication for community engagement

Capital costs include the cost of vehicle rental, the annualised costs of non-rented vehicles used and the cost of spray guns. The costs were adjusted for time use as the vehicles were used for other programmes as well. We estimated that these vehicles were used 27% of the time for the mosquito collection phase. In terms of the share of each cost group, the majority of the recurrent costs were personnel-related costs (21,370.04 USD) with mosquito collectors costing the most (54.5%) and supervisors costing the least (17.3%). A bulk of the capital costs (88.7%) were related to transportation (Fig. 3b).

## Discussion

Transmission assessment surveys (TAS) to determine whether or not MDA can be stopped [23] are based on prevalence of infection in the human population. This has no real transmission component involving vectors due to the ease of sampling human populations. Xenomonitoring surveys, on the other hand, are considered expensive, requiring large number of mosquitoes and limited technical expertise [2]. Notwithstanding the limitations associated with xenomonitoring, a recent study in Togo [24] using molecular xenomonitoring for post-validation surveillance of lymphatic filariasis demonstrated the feasibility of its application on a larger scale. To overcome the above challenges, various tools and approaches are being developed, including laboratory and field practical methodologies [25, 26]. In this study, we evaluated the use of CVCs for the purposes of assessing their usefulness in collecting large numbers of mosquitoes at low costs. Our results indicate that CVCs may indeed be useful in xenomonitoring activities for lymphatic filariasis elimination programmes. The costs incurred for collection of mosquitoes was significantly lower compared to using a research team. Dorkenoo et al. [24] also demonstrated in their study a lower cost in using CVCs for xenomonitoring in post-validation surveillance of lymphatic filariasis in Togo. Moreover, CVCs may promote active community participation and enhance ownership of vector control activities for the control and monitoring of vector-borne diseases [27].

It has been argued that implementing community-based mosquito collection schemes present two important challenges. The first challenge is the selection of traps that are safe, practical and convenient for CVCs to apply them reliably in the absence of daily supervision. The second challenge is the need for an independent quality assurance of this unsupervised surveillance process, so that the accuracy and limitations of the derived data can be quantified as a prerequisite to critical interpretation [12]. The use of CVCs may require programmatic guidelines and procedures so as to streamline the process and protocols for mosquito collection.

In the rainy season, mosquito densities increased compared to the dry season. This may expose the collectors to more infectious mosquito bites [28]. As such, alternatives to the human landing collections, such as the human-baited double net traps [29], will provide protection to the collectors while allowing large numbers of mosquitoes to be collected. Proper training in mosquito collection methods will also be required. The differences in the number of mosquitoes between the southern and northern communities may be attributed to the environmental characteristics of the areas [30]. However, the effectiveness of the trapping method may indicate the need to consider different sample collection methods in different areas.

In this study, the amount paid to the collectors was negotiated based on the number of days and activities to be undertaken. While the cost per collector sampling per month (15.17 USD) was much lower than the approximate 70.00 USD reported in a community based scheme in Zambia [12], we believe the mean cost per person could greatly be reduced if lesser number of collection methods are implemented and a community ownership model is employed. The use of a CVC strategy could further be implemented as part of monitoring and evaluation and TAS activities, as lymphatic filariasis control and elimination programmes spend a considerable amount of time in disease endemic communities every year. Thus, integrating the CVC strategy with ongoing lymphatic filariasis programme activities will further reduce the transportation costs associated with the implementation of xenomonitoring surveys.

There were a couple of limitations to this study. First, the validation was done only on two occasions (both dry and wet season), and the environmental variables in each community may have influenced the numbers of mosquitoes collected by the CVCs. Nonetheless it is assumed that the results are representative of the collectors and trap performance in the study. Secondly, the study failed to assess the views of the CVCs and community members towards the implementation of this strategy. This would have provided important information on the community acceptability and feasibility of upscaling this strategy. Lastly, the study was unable to disaggregate the current cost based on community and on method of mosquito collection. Future research should be able to attribute the costs to the main method of collection and adjust for community variations in costs.

## Conclusions

This study showed that the use of CVCs for lymphatic filariasis xenomonitoring activities is feasible and may be a useful strategy in overcoming the challenges associated with sampling large numbers of mosquitoes and evaluating the spatio-temporal patterns of lymphatic filariasis vector transmission indices. It also showed that the cost for vector collection may be greatly reduced, enabling a wide rollout of this strategy for lymphatic filariasis xenomonitoring activities. Further evaluation needs to be undertaken to assess the criteria for selecting and motivating CVCs, the acceptability of CVCs for monitoring disease programmes, knowledge, attitude and practices of vector collectors, and epidemiological relevance of this strategy for lymphatic filariasis xenomonitoring activities.

## Abbreviations

CDD: Community drug distributors
CHPS: Community-based health planning and services
CVC: Community vector collectors
DEC: Diethylcarbamazine
ESPEN: Expanded Special Project for Elimination of Neglected Tropical Diseases
GHS: Ghana Health Service
GPELF: Global Programme to Eliminate Lymphatic Filariasis
HLC: Human landing collections
IDA: Ivermectin plus diethylcarbamazine (citrate) plus albendazole
IEC: Information, education and communication
MDA: Mass drug administration
PSC: Pyrethrum spray collections
TAS: Transmission assessment survey
VAL: Validation
WET: Window exit trap
WHO: World Health Organization
